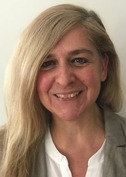# Peptide‐Based Molecular Strategies To Interfere with Protein Misfolding, Aggregation, and Cell Degeneration

**DOI:** 10.1002/anie.201906908

**Published:** 2019-11-28

**Authors:** Valentina Armiento, Anna Spanopoulou, Aphrodite Kapurniotu

**Affiliations:** ^1^ Division of Peptide Biochemistry TUM School of Life Sciences Technische Universität München Emil-Erlenmeyer-Forum 5 85354 Freising Germany; ^2^ Current address: Coriolis Pharma Research GmbH Fraunhoferstrasse 18B 82152 Planegg Germany

**Keywords:** Alzheimer's disease, amyloid inhibitors, anti-amyloid drugs, peptides, protein aggregation

## Abstract

Protein misfolding into amyloid fibrils is linked to more than 40 as yet incurable cell‐ and neurodegenerative diseases such as Alzheimer's disease, Parkinson's disease, and type 2 diabetes. So far, however, only one of the numerous anti‐amyloid molecules has reached patients. This Minireview gives an overview of molecular strategies and peptide chemistry “tools” to design, develop, and discover peptide‐based molecules as anti‐amyloid drug candidates. We focus on two major inhibitor rational design strategies: 1) the oldest and most common strategy, based on molecular recognition elements of amyloid self‐assembly, and 2) a more recent approach, based on cross‐amyloid interactions. We discuss why peptide‐based amyloid inhibitors, in particular their advanced generations, can be promising leads or candidates for anti‐amyloid drugs as well as valuable tools for deciphering amyloid‐mediated cell damage and its link to disease pathogenesis.

## Graphical abstract

**Aberrant protein aggregation** in amyloid fibrils is linked to many devastating and thus far incurable cell‐degenerative diseases such as Alzheimer's disease. However, only one of the numerous anti‐amyloid candidates has reached the clinic. This Minireview discusses peptide‐based molecular strategies and peptide chemistry “tools” for the design, development, and discovery of peptides as leads for anti‐amyloid drugs.

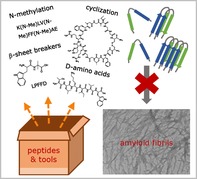

## 1 Introduction

### 1.1 Protein Misfolding, Amyloid Formation, and Cell and Neurodegenerative Diseases

**Figure 1:**
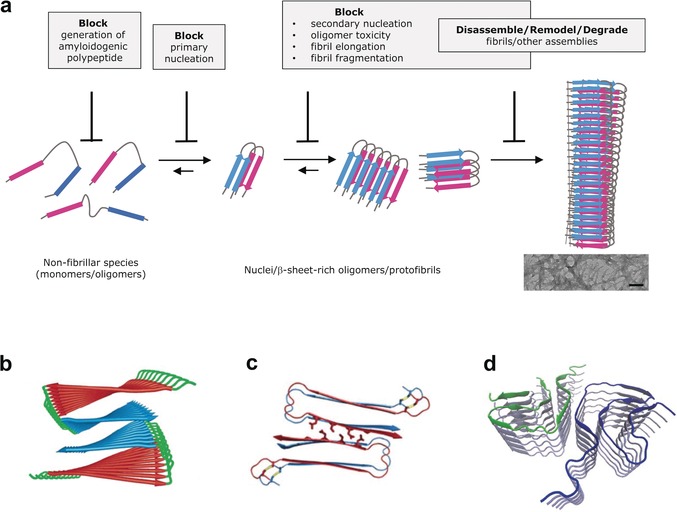
a) Amyloid self‐assembly and molecular strategies for interference and b–d) structural models of amyloid fibrils. b) Model of Aβ40 fibrils based on ssNMR studies by the Tycko group (Copyright (2006) National Academy of Sciences).^6^ c) The IAPP fibril model of Eisenberg et al. based on crystal structures of IAPP segments (reproduced with permission from Wiley (copyright)).^7^ d) Structure of the αSyn fibril core αSyn(38–95) determined by cryo‐EM studies by the Stahlberg group (PDB: 6H6B).^8^ TEM image in (a): scale bar 100 nm.

Protein misfolding and aggregation into amyloid fibrils is linked to the pathogenesis of more than 40 devastating cell‐ and neurodegenerative diseases.^1^ Prominent examples are Alzheimer's disease (AD), Parkinson's disease (PD), Huntington's disease (HD), type 2 diabetes (T2D), prion protein (PrP) related encephalopathies, and many other amyloidoses.^1^ In these diseases, a specific polypeptide or protein misfolds from a normally soluble, nonfibrillar nontoxic state into a β‐sheet‐rich ensemble of cytotoxic aggregates and amyloid fibrils (Figure 1).^1^, ^2^ For example, amyloid plaques in brains of AD patients contain the 40‐ and 42‐residue amyloid‐β polypeptides Aβ40 and Aβ42 as well as neurofibrillary tangles of the 352–441‐residue segments of the microtubule‐associated protein tau. In contrast, amyloid deposits in brains of PD patients contain the 140‐residue α‐synuclein (αSyn), and T2D pancreatic amyloid deposits contain the 37‐residue islet amyloid polypeptide (IAPP).^1^ The amyloidogenic polypeptides exhibit distinct physiological functions: for example, Aβ is likely involved in protection of the central nervous system, αSyn regulates synaptic function, and IAPP is a neuropeptide hormone regulator of glucose homeostasis.^3^

The process of amyloid formation is believed to be a primary event in cell degeneration and amyloid disease pathogenesis.^4^ Amyloid fibrils derived from all polypeptides have similar morphology, that is, diameters of 7–20 nm, lengths up to several micrometers, and they consist of protofilaments.^1^, ^2^ They exhibit a “cross‐β” structure, that is, their spines consist of β‐sheets arranged in parallel to the fibril axis with the strands running perpendicular to it (Figure 1).^2^ In the last 10–20 years, results from (cryo‐)electron microscopy (EM), X‐ray microcrystallography, solid‐state NMR spectroscopy (ssNMR), and other biophysical studies have provided key insights into some amyloid structures (Figure 1).^2^ Cell‐damaging properties are ascribed both to amyloid fibrils and to transient prefibrillar oligo‐/multimers. Aggregate toxicity is likely mediated by common mechanisms and caused by both direct effects on the cell membranes and indirect ones, such as inflammation and cell‐to‐cell transmission.^1^, ^5^

Amyloid self‐assembly proceeds by the following mechanism: 1) nucleation‐dependent polymerization, 2) nucleation‐dependent conformational conversion, 3) downhill polymerization, and 4) native‐like aggregation.^1^, ^4^ Key molecular events include: primary nucleation, that is, formation of the nucleus, secondary nucleation, fibril elongation, and fibril fragmentation.^1^, ^4^ Amyloid formation is controlled by various biomolecular interactions, including interactions of amyloid polypeptides with other proteins, for example, chaperones, and through cross‐amyloid interactions.^5^, ^9^ Prominent cross‐amyloid interactions are Aβ with tau, PrP, αSyn, TTR, insulin, or IAPP as well as IAPP with insulin or αSyn.^10^ These can accelerate or suppress amyloidogenesis depending on the nature and structure/assembly state of the partners.^10^, ^11^ For example, Aβ fibrils cross‐seed IAPP fibrillogenesis, whereas interactions of nonfibrillar Aβ and IAPP species yield nonfibrillar and nontoxic hetero‐oligomers which attenuate fibrillogenesis.11c, 12 Cross‐amyloid interactions may thus link different diseases to each other, for example, AD with T2D, AD with PD etc.^5^, ^10^, 11c, 12b

### 1.2 Inhibition of Amyloid Formation: Concepts and Molecules

Over the past 25 years, numerous anti‐amyloid molecules have been reported.^1^, ^4^ Most of them were evaluated with in vitro assays; studies in animal models were reported only for some of them.^4^, ^13^ Most of these agents belong to the following classes: 1) antibodies/proteins, 2) small organic molecules, and 3) peptides and peptidomimetics.^4^, ^13^, ^14^ Several promising anti‐amyloid drug candidates have been and are currently being tested in clinical studies.14c For example, blocking amyloid formation of Aβ or tau in AD is the target of more than half of the agents in phase III clinical trials.14c However, so far only one of the anti‐amyloid drug candidates—the small molecule “Tafamidis” developed by Kelly and co‐workers, which inhibits transthyretin (TTR) amyloidogenesis (familial amyloid polyneuropathy (FAP) treatment)—has reached the clinic.^4^

The following molecular strategies have been developed to interfere with amyloid formation (Figure 1):

1. block generation of the amyloidogenic protein (e.g. by proteolytic processing of a precursor),
2. block primary nucleation, for example, by monomer stabilization/sequestering,
3. block secondary nucleation, for example, by binding on the surface of protofibrils/fibrils/oligomers,
4. block fibril elongation, for example, by capping, and
5. remodeling/disassembly/degradation of fibrils or other assemblies.

The rational design of potent inhibitors of amyloid formation is, however, a great challenge. Major reasons are:

1. the lack of a defined structure of the target(s) due to the often intrinsically disordered nature of amyloidogenic proteins and the transient nature of their on‐/off‐pathway assemblies,
2. the dynamic nature of self‐assembly; for example, inhibitor binding to the “wrong” species could cause an increase in the amount of cytotoxic species because of a shift in the self‐assembly equilibria,
3. the plasticity of amyloid assemblies and large size of the involved surfaces, and
4. the fact, that amyloid self‐assembly and related cell‐toxic pathways often occur at low nanomolar protein concentrations; high affinity inhibitors are thus required.

Moreover, promising leads for anti‐amyloid drugs must also fulfill several other requirements such as target specificity, solubility, stability to proteolytic degradation, suitable pharmacological properties (ADME), and, in some cases, cell or blood–brain barrier (BBB) permeability.

In this Minireview we discuss peptide‐based molecular strategies to interfere with amyloid self‐assembly. We focus on cases that exemplify the use of peptide chemistry strategies and “tools”. Strategies to interfere with the generation of the amyloidogenic polypeptides (mainly by small molecules) are not discussed. As the targets of most reported peptide inhibitors have been Aβ (AD) and IAPP (T2D), with AD being the most common form of dementia and T2D reaching epidemic levels globally, the Minireview refers mainly to them.

## 2 Peptides as Therapeutics and Candidates for Anti‐Amyloid Drugs

Until the end of the 20th century, peptides were considered to be less suitable than small molecules or antibodies as drugs.^15^ Their disadvantages were believed to outweigh their advantages. Major weaknesses are low proteolytic stability, rapid clearance, poor bioavailability/oral availability, and possible chemical/physical instability.^15^ On the other hand, high efficacy, high selectivity or specificity, and high potency are their major strengths.^15^

Over the past two decades, however, peptide drugs have seen a renaissance.^15^, ^16^ Today, more than 60 peptide drugs are on the market—more than 150 are in the clinic and more than 200 in preclinical development. Word‐wide sales exceed $50 billion.^15^ In addition to various failures of small‐molecule drug candidates and the extremely high costs of antibody‐based therapies, major advances in peptide and medicinal chemistry played an important role in the change of perception toward peptide‐based drugs.^15^, ^16^, ^17^

Peptides are an attractive alternative to small molecules and antibodies as anti‐amyloid drugs. In fact, small molecules lack the large surfaces often required for potent amyloid inhibitors and are often nonspecific, while antibodies are characterized by very high production costs, no/low cell‐membrane or BBB permeability, and potential immunogenicity. In particular, conformationally constrained small‐/medium‐sized peptides, such as (macro)cyclic peptides, can combine several of the advantages of biopharmaceuticals (biologics) or antibodies, such as high potency and target selectivity with stability, bioavailability, low/no immunogenicity, and the low production costs of small molecules.^18^ Moreover, macrocyclic peptides often exhibit large surface areas and cell or BBB permeability, which further adds to their potential suitability as anti‐amyloid leads.18c

## 3 Design and Discovery of Peptide Drugs: Approaches and Peptide Chemistry “Toolbox”

**Figure 2:**
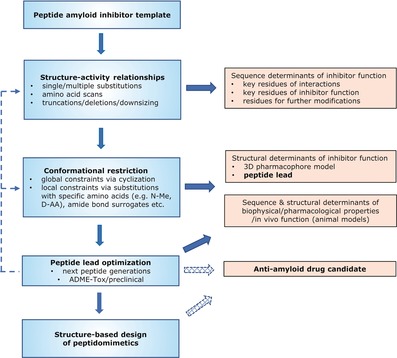
A general approach to design and develop peptide‐based anti‐amyloid drug candidates (scheme inspired from Figure 1 from Ref. 19b).

The design, optimization, and discovery of peptide drugs are based on hierarchical strategies developed early on by pioneers of peptide science, and by using several peptide chemistry “tools” such as (Figure 2):^15^, ^16^, ^17^, 18b, 19

1. single/multiple substitutions or “scans” (e.g. Ala scan) with native or non‐native amino acids,
2. sequence truncations/deletions/downsizing,
3. global/local conformational restriction strategies involving a) peptide cyclization (e.g. head‐to‐tail, side‐chain‐to‐side‐chain etc.) by various different chemical approaches/linkers (e.g. disulfides, cysteine stapling, lactam bridges, hydrocarbon stapling, triazoles), b) substitutions with special conformationally restricted amino acids (e.g. Cα‐alkylated, N‐methylated, d‐amino acids), or c) peptide‐bond replacement with “surrogates” (e.g. reduced peptide bonds, retro‐, retro‐/inverso‐),
4. coupling with specific “tags” (e.g. solubility or cell‐permeable ones), and
5. N‐/C‐terminal and other modifications (e.g. acylation, PEGylation etc).^15^, ^16^, ^17^, 18b

## 4 Peptide‐Based Molecular Strategies To Inhibit Amyloid Formation

**Figure 3:**
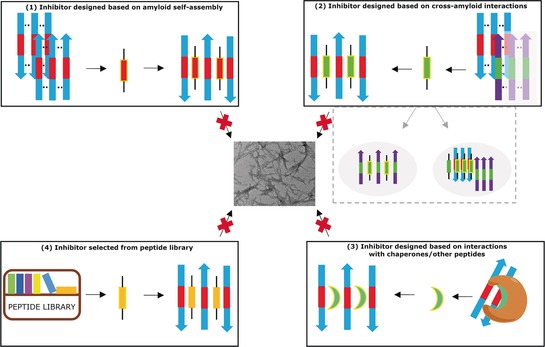
Peptide‐based molecular strategies (1)–(4) to inhibit amyloid formation. The dashed box under (2) indicates additional inhibitor functions when the same “hot segments” of the cross‐interaction partner mediate both its cross‐interactions and its self‐assembly.

Most peptide inhibitors have been devised on the basis of four strategies (Figure 3): In the first three, inhibitor design is based on molecular recognition principles of amyloid self‐assembly (1), cross‐amyloid interactions (2), or interactions with chaperones or other non‐amyloidogenic polypeptides (3); in the fourth strategy, inhibitors are discovered using combinatorial libraries and optimized with peptide chemistry tools. Here we focus on strategies (1) and (2).

### 4.1 Peptide Inhibitors Designed on the Basis of Amyloid Self‐Assembly

#### 4.1.1 Inhibitors Derived from Amyloid Self‐Recognition Segments

**Figure 4:**
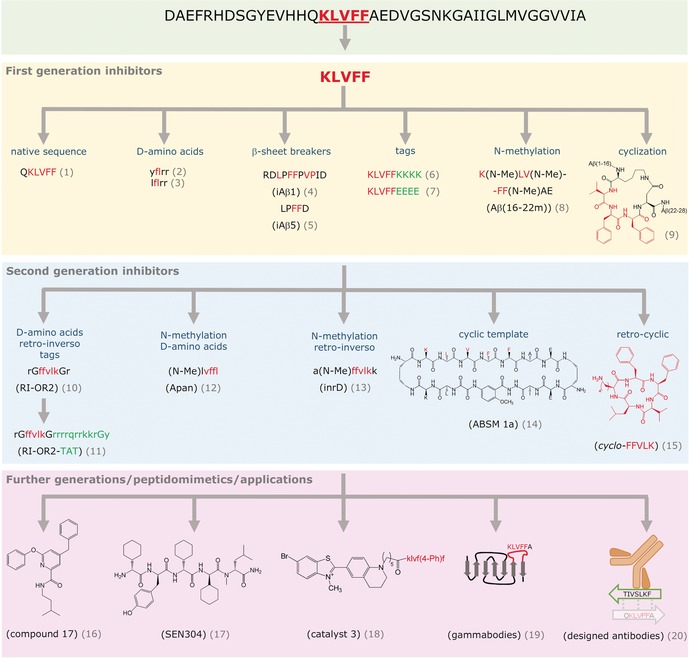
Selected KLVFF‐based inhibitors of Aβ amyloid self‐assembly and related peptide chemistry tools. Green box: Aβ42 sequence; amyloid core KLVFF in red; yellow box: 1st generation peptide inhibitors **1**–**9**;^21^, ^22^, ^23^, ^24^, 25b, 27a, 29 blue box: 2nd generation peptide inhibitors **10**–**15**;^30^ pink box: further peptide generations, peptidomimetics, and applications.27e, 30f, 31 Peptide N‐/C‐termini, not shown; inhibitor abbreviations used in original reports in brackets; KLVFF, red; tags, green.

This strategy has been the most commonly applied one.^13^, ^20^ Inhibitors are derived from or contain a self‐recognition or “amyloid core” region after suitable modification(s) with peptide chemistry tools (Figures 2 and 3). The strategy is exemplified by discussing mainly amyloid inhibitors of Aβ40(42) derived from its self‐recognition segment Aβ(16–20) (Figure 4).

The proof‐of‐principle was provided early on. In 1996, Tjernberg et al. used peptide arrays and identified Aβ(16–20) or KLVFF as a minimum self‐recognition sequence of Aβ40 (Figure 4).^21^ It is noteworthy that at that time structural models of Aβ fibrils did not exist. Moreover, Tjernberg et al. found that Aβ(15–20) or QKLVFF (**1**; Figure 4) suppressed Aβ40 fibrillogenesis. However, amyloid core sequences are usually intrinsically highly amyloidogenic and poorly soluble. Thus, in the same year Soto et al. reported short, KLVFF‐derived peptides containing the “β‐sheet breaker” Pro; these peptides were non‐amyloidogenic and able to inhibit Aβ40(42) fibrillogenesis (e.g. **4** and **5**; Figure 4).^22^ The pentapeptide LPFFD (**5**; Figure 4) strongly suppressed Aβ40(42) fibrillogenesis both in vitro and in a rat model in vivo, thus becoming one of the earliest peptide leads for anti‐amyloid drugs.^22^, ^23^ In addition, analogues containing d‐amino acids were synthesized, which afforded inhibitors with improved proteolytic stability (e.g. **2** and **3**; Figure 4).^22^, ^24^ By using a different peptide chemistry tool, Kiesling, Murphy, and co‐workers designed KLVFF‐containing peptides (e.g. **6** and **7**; Figure 4) linked to “disruptive elements”, for example, oligo‐Lys or ‐Glu tags, as potent inhibitors of Aβ40 cytotoxicity.^25^ These studies paved the way for various KLVFF‐derived peptides, peptidomimetics, and Aβ‐binding proteins. Moreover, similar concepts were used for other amyloid polypeptides (Figure 4).^13^, 14b, 26

One broadly applied peptide chemistry tool is the N‐methylation of amide bonds.^27^ The N‐methylation of amide bonds restricts peptide conformation and the ability to propagate β‐sheets, improves peptide solubility and proteolytic resistance, and may confer membrane or BBB permeability.^28^ The earliest amyloid targets were Aβ and IAPP. In the case of Aβ, the Doig, Meredith, Findeis, and Giralt groups reported N‐methylated analogues of Aβ self‐recognition segments to be inhibitors of Aβ40(42) amyloid in the early 2000s (Figure 4).27a, 27f, 32 In addition, the Giralt group applied the retro‐inverso approach to N‐methylated peptides, which yielded an inhibitor exhibiting high proteolytic stability (**13**; Figure 4).30d As a consequence of their favorable properties, some N‐methylated peptides entered (pre)clinical trials (e.g. **12**, **17**; Figure 4); however, none of them has yet found clinical application.^13^, 27e, 30a, 30b, 32b In the case of IAPP, our group applied N‐methylation to peptides containing its amyloid core segment NFGAIL (IAPP(22–27)).27b, 33 The doubly N‐methylated hexapeptide NFGAIL‐GI was non‐amyloidogenic and effectively suppressed the self‐assembly and cytotoxicity of IAPP amyloid.27b, 33 Moreover, several doubly N‐methylated full‐length IAPP analogues were designed which combined high solubility, non‐amyloidogenicity, and IAPP‐like bioactivity with nanomolar inhibitor function in the IAPP cytotoxic self‐assembly.27d, 34

Another tool applied to Aβ early on was conformational restriction through cyclization. In 2003, we showed that conformational restriction of the Aβ amyloid core segment VFF or Aβ(18–20) within Aβ(1–28) through side‐chain‐to‐side‐chain cyclization yielded the non‐amyloidogenic analogue **9** (Figure 4). Importantly, **9** blocked the amyloidogenesis of Aβ(1–28) and Aβ40; thus, cyclization converted an amyloid sequence into an amyloid inhibitor.^29^ In 2011 to 2012, an innovative “cyclic template” concept was reported by the Nowick and Eisenberg groups.^35^ Macrocyclic peptides called “amyloid β‐sheet mimics” (ABSMs) were designed as potent amyloid inhibitors of tau, Aβ, IAPP, or αSyn.30e, 35 The ABSMs consisted of a “recognition” and a “blocker” β‐strand. The former one displayed an amyloid core segment, conferring target specificity, while the “blocker” β‐strand contained the unnatural residue “Hao”, a β‐sheet blocker.30e, 35 The KLVFFAE‐containing **14** (Figure 4) was suggested to inhibit the formation and toxicity of Aβ42 amyloid by sequestering Aβ42 oligomers into alternate pathways.30e

A good example of how cyclization in combination with peptide chemistry tools and rational design may yield small‐molecule peptidomimetics as amyloid inhibitors was provided by the Kanai and Sohma groups.30f Starting with head‐to‐tail‐cyclized Aβ(15–20) or cyclo‐KLVFF, which were found to suppress Aβ42 fibrillogenesis, enantio and retro‐enantio analogues were synthesized (e.g. **15**; Figure 4). Small‐molecule peptidomimetics were then designed and, indeed, two of them suppressed Aβ42 fibrillogenesis (e.g. **16**; Figure 4).30f

Amyloid core sequences were also used as tags for the selective and high‐affinity recognition of amyloid polypeptides.^13^ For example, the Sohma and Kanai groups presented an innovative concept, called “target‐sensing catalyst activation” (TaSCAc), to generate switchable photooxygenation catalysts of Aβ42 amyloid. These molecules consisted of an amyloid‐detecting fluorescent probe, a thioflavin‐based photooxygenation moiety, and a KLVFF analogue for Aβ‐binding (e.g. **18**; Figure 4). The concept may be applicable to other amyloid polypeptides; however, its potential applicability in treating amyloid diseases is still unclear.31a, 36

In a further development, amyloid core sequences, for example, KLVFF, were used to generate antibody‐based inhibitors (Figure 4).31b, 31c, 37 Amyloid core segments or complementary peptides were grafted into an antibody‐derived scaffold (“gammabodies”) or an antibody by the Tessier and Vendruscolo groups, respectively (**19** and **20**; Figure 4). Potent sequence‐ and/or conformation‐specific amyloid inhibitors of Aβ, IAPP, or α‐synuclein were thereby generated.31b, 31c, 37

#### 4.1.2 Inhibitors Designed on the Basis of Molecular Recognition Features of Amyloid Self‐Assembly

**Figure 5:**
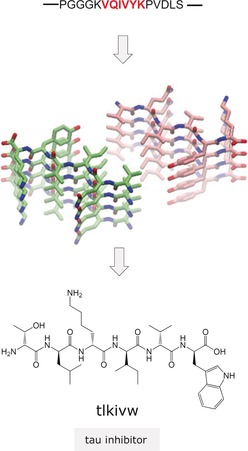
Structure‐based inhibitor design approach reported by the Eisenberg and Baker groups and exemplified by the design of a tau amyloid inhibitor based on the steric zipper‐based (PDB: 5k7n) all‐d‐peptide tau segment.^38^

A major development has been the structure‐based and computer‐aided rational design inhibitor approach reported by the Eisenberg and Baker groups about eight years ago (Figure 5).^38^ The approach uses atomic structures of crystals of short amyloid‐forming segments as templates and Rosetta software to design short peptides that cap fibril ends. Previously, the Eisenberg group had shown that crystal structures of short amyloid segments share a common “steric zipper” motif, that is, the β‐sheets interact by side‐chain interdigitation, as seen in amyloid fibrils.^39^ Inhibitor design was based on the hypothesis that short amyloid core sequences form the same steric zippers in their crystals and in the fibrils of their full‐length proteins. The proof‐of‐principle was provided by the successful design of an all‐d‐hexapeptide as an inhibitor of tau amyloidogenesis (Figure 5).^38^ Since then, several potent peptide inhibitors of tau and other proteins including TTR, IAPP, and Aβ42 have been designed. This approach should greatly speed up the discovery of peptide leads for anti‐amyloid drugs.^40^

**Figure 6:**
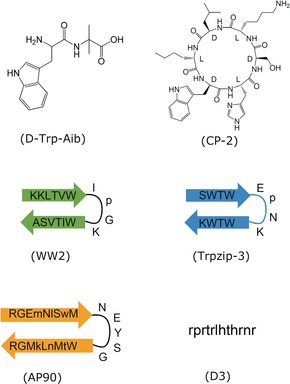
Peptide inhibitors designed on the basis of general molecular recognition principles of amyloidogenesis or selected by combinatorial library approaches (inhibitor names in original reports in brackets).^41^, ^42^, ^43^, ^44^

Ten years ago, the Gazit group reported an innovative minimalistic approach for the design of peptide inhibitors: based on the key role of aromatic interactions and β‐breaker elements in amyloidogenesis, 40 short peptides consisting of these two elements were designed.^41^ The dipeptide d‐Trp‐Aib (Figure 6) effectively suppressed the formation of Aβ42 amyloid fibrils and cytotoxic oligomers, likely through binding early nontoxic oligomers. Furthermore, d‐Trp‐Aib resulted in less amyloid deposits and improved cognitive performance in an AD mouse model. These and other features, for example, good oral bioavailability and BBB crossing, made d‐Trp‐Aib a promising candidate for anti‐amyloid drugs; notably, this dipeptide also suppressed amyloidogenesis of IAPP, αSyn, and calcitonin.^42^

A good example of a cyclic peptide inhibitor discovery approach was provided by the Rahimipour group.^43^ Capitalizing on the strong self‐assembly propensity of d,l‐α‐cyclic peptides and the structural similarity of their tubular assemblies with amyloids, a focused library of cyclic d,l‐α‐hexapeptides was designed; CP2 (Figure 6), one of the peptides, also suppressed amyloidogenesis of Aβ40(42), αSyn, and the tau amyloid core hexapeptide AcPHF6.^45^ Inhibition was likely mediated by the ability of CP‐2 aggregates to bind amyloidogenic oligomers and to penetrate cell membranes. Such cyclic peptides could become leads for anti‐amyloid drugs should their intrinsic cell penetration ability cause no adverse effects.

The last example refers to designed prestructured β‐hairpins bearing no sequence similarity to their targets, but mimic some of their structural features (Figure 6). These were reported by the Andersen and Daggett groups and comprise short β‐hairpin or “tryptophan zipper (Trpzip)” based peptides (e.g. WW2 and Trpzip‐3; Figure 6).44a, 44b, 46 Notably, some of them suppressed amyloidogenesis of two polypeptides simultaneously, that is, of IAPP and αSyn or of Aβ42 and TTR.44a, 44b, 46 Interactions of inhibitors with prefibrillar species of the various different amyloid polypeptides, which likely share some structural similarity, were suggested to underlie their inhibitory effects.44a Furthermore, the Daggett group designed AP90, an α‐hairpin containing alternating d‐ and l‐residues (Figure 6).44c, 47 AP90 and related α‐sheet‐forming peptides suppressed the amyloid self‐assembly of three different polypeptides, that is, Aβ42, TTR, and IAPP. Notably, the inhibitory activity was independent of the inhibitor sequence and was suggested to be mediated by interactions with α‐sheet‐rich cytotoxic Aβ42 oligomers, which were proposed to be key intermediates of amyloid self‐asssembly.44c, 47

It appears, thus, that peptide‐based inhibitors displaying general amyloid recognition features can also be multitarget inhibitors, similar to other classes of molecules.^48^ Their suitability as leads for anti‐amyloid drugs by combining multitarget activity with target selectivity is still to be evaluated; however, their selectivities should outperform those of small molecules.^15^

### 4.2 Inhibitors Designed on the Basis of Cross‐Amyloid Interactions

According to this more recently developed strategy, the inhibitor is derived from an amyloidogenic polypeptide which cross‐interacts with the target polypeptide; the full‐length interaction partner or its “hot segments” can be used (Figure 3). In some cases, these segments also mediate the self‐assembly of the partner; their use may additionally then yield both 1) cross‐amyloid inhibitors, that is, inhibitors of amyloid self‐assembly of both polypeptides, and 2) inhibitors of a potentially harmful cross‐amyloid interaction (e.g. cross‐seeding). Such properties can strongly expand the functional profile of the inhibitor (Figure 3).11c, 11d, 27d, 40c, 49

#### 4.2.1 Inhibitors Designed on the Basis of the IAPP‐Aβ Cross‐Interaction

**Figure 7:**
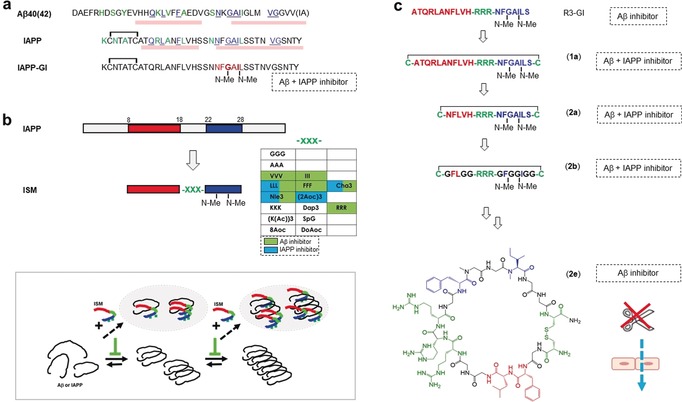
(Cross‐)amyloid nanomolar peptide inhibitors of Aβ40(42) and/or IAPP designed on the basis of the IAPP‐Aβ cross‐interaction. a) Aβ40(42), IAPP (similar residues, green; identical, blue and underlined; pink bars: residues in β‐strand), and IAPP‐GI (IAPP amyloid core NFGAIL, red).11c, 27d, 34 b) Design concept, inhibitory effects, and suggested mechanism of the inhibitor function of ISMs.49a c) Minimalistic design concept, sequences, and functions of the MCIPs.49b

The high‐affinity cross‐interaction between IAPP and Aβ was the first to become exploited for the design of cross‐amyloid inhibitors (Figure 7 a).11b, 11c Considering the high sequence and structural similarity of the two polypeptides, we wondered whether our non‐amyloidogenic N‐methylated IAPP analogues, which inhibited the amyloid self‐assembly of IAPP, might also interfere with Aβ amyloidogenesis (Figure 7 a).11c, 27d, 50 In fact, IAPP‐GI and the other analogues turned out to be nanomolar inhibitors of the cytotoxic self‐assembly of Aβ40; IAPP‐GI was thus the first reported peptide cross‐amyloid inhibitor of both IAPP and Aβ40(42).11c, 27d Inhibition was mediated by nonfibrillar/nontoxic inhibitor‐Aβ40 hetero‐oligomers and by fibril remodeling/disassembly, as also found for effects on IAPP amyloidogenesis.11c, 27d, 34

Our more recent (cross‐)amyloid inhibitor designs are the so called “interaction surface mimics” or ISMs.49a This series of IAPP‐derived 21‐residue peptides were designed to mimic putative IAPP self‐ and/or Aβ cross‐interaction surfaces. ISMs were found to be nanomolar cross‐ or target‐selective amyloid inhibitors of Aβ40(42) and/or IAPP (Figure 7 b). Importantly, some of them also blocked the cross‐seeding of IAPP by Aβ40 fibrils, a possible link between the two diseases.^5^, 11b, 12b ISMs are thus good leads for anti‐amyloid drugs in AD, T2D, or both diseases.12b, 51 Their design was based on the finding that IAPP uses the same two hot segments for both its self‐ and its cross‐amyloid interaction with Aβ40(42) (Figure 7 b).^50^ ISMs were generated by linking the “hot segments” (in the native or N‐methylated form) through structurally biased linkers; importantly, the linker determined the inhibitor potency and target selectivity.49a Inhibition was mainly mediated by the high‐affinity binding of ISMs or their aggregates to prefibrillar Aβ40(42) or IAPP species and their sequestration into amorphous/nontoxic heteroaggregates (Figure 7 b).

Most recently, ISM R3‐GI (Figure 7 c) was used as a template to design novel macrocyclic peptides as potent amyloid inhibitors.49b These macrocyclic inhibitory peptides (MCIPs) mimic functional (inhibitory) IAPP interaction surfaces while maintaining minimal IAPP‐derived recognition elements (Figure 7 c).49b, 52 We used a minimalistic approach and various peptide chemistry tools for their design. The 17‐residue **2 b** containing only four IAPP‐derived residues was a nanomolar inhibitor of the cytotoxic self‐assembly of both Aβ40(42) and IAPP (Figure 7 c). However, **2 b** was rapidly degraded by serum proteases. Systematic l‐/d‐residue exchange led to the generation of MCIP **2 e**, a selective nanomolar inhibitor of Aβ40(42) with high proteolytic stability in human serum (Figure 7 c). As **2 e** also crossed the BBB in a human cell model, it is a promising candidate for AD anti‐amyloid drugs.

#### 4.2.2 Inhibitors Designed on the Basis of the IAPP–Insulin Interaction

In 1996, Westermark et al. reported that insulin inhibits the amyloid self‐assembly of IAPP.11a Several years later, the Gazit group identified binding sites and short peptides of the B chain of insulin which suppressed IAPP fibrillogenesis, and the Eisenberg group suggested that IAPP(8–27) forms the interaction surface of IAPP with itself and with insulin.^7^, ^53^ As the non‐native aggregation of insulin complicates its biomedical application, we wondered whether IAPP‐GI might interfere with the aggregation of insulin. In fact, IAPP‐GI was found to be a nanomolar inhibitor of non‐native insulin aggregation.11d Importantly, as expected for a native interaction partner, IAPP‐GI–insulin interactions do not interfere with insulin function.11d Thus, the functional profile of IAPP‐GI comprises potent anti‐amyloid activity toward Aβ, IAPP, and insulin in combination with IAPP‐like bioactivity, which makes this peptide a promising lead for anti‐amyloid drugs in both AD and T2D, also in combination with insulin‐based treatments. Clinical trials on insulin‐based treatments in AD are in progress.11d, 14c, 51

#### 4.2.3 Inhibitors Designed on the Basis of the TTR–Aβ Interaction

**Figure 8:**
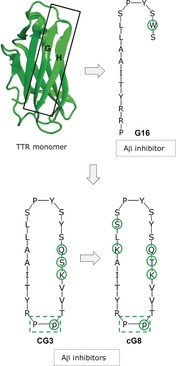
Peptide inhibitors of Aβ40 amyloid self‐assembly designed on the basis of the TTR‐Aβ cross‐interaction (TTR structure from PDB 1DVQ; substitutions in inhibitors in green circles; β‐turn‐stabilizing dipeptide in dashed green box).^56^

The TTR‐Aβ cross‐interaction effectively suppresses the deposition of Aβ amyloid.10a, 54 In 2014, Murphy and co‐workers reasoned that peptides mimicking TTR strand G and parts of strand H, namely, the Aβ‐binding site of TTR, might inhibit the self‐assembly of Aβ amyloid.^55^ In fact, the 16‐residue linear peptide G16, a Y116W mutant of TTR(102–117), bound Aβ40 and suppressed its cytotoxicity (Figure 8).^55^ To improve its properties, more residues from strand H were added, the backbone cyclized, and the β‐hairpin stabilized. The resulting cyclic CG3 was indeed more effective and its further optimization yielded the more potent cG8 (Figure 8).^56^, ^57^ These “structural mimics” of the Aβ binding region of TTR may act by redirecting Aβ40 into large nonfibrillar aggregates which are more easily degraded than Aβ40 fibrils.^56^, ^57^ If applicable to the in vivo situation, Aβ40 clearance would become facilitated, which renders these peptides promising leads for AD anti‐amyloid drugs.

### 4.3 Inhibitors Based on Interactions with Chaperones and Other Binding Proteins

Peptides from functional regions of chaperones targeting amyloid self‐assembly could act as templates for designing highly effective mini‐chaperones (Figure 3).9c, 58 For example, αΒ‐crystallin contains many segments capable of suppressing the fibrillogenesis of Aβ42 and/or α‐synuclein.^58^, ^59^ However, the dynamic and often promiscuous nature of interactions and functions of α‐crystallins may complicate inhibitor design.9c Thus, the applicability of this approach is yet to be demonstrated. Another example is the BRICHOS domain of the chaperone Bri2, a highly effective amyloid inhibitor of both Aβ42 and IAPP. BRICHOS peptides could be useful scaffolds for inhibitor design.^60^

A good example of a peptide designed on the basis of a cross‐interaction and aiming at blocking this interaction is Aβ12‐28P; this is an analogue of Aβ(12–28) which mediates the Aβ‐apoE4 interaction. Blocking this interaction with Aβ12‐28P or its optimized analogue CPO_Aβ17‐21 P, was recently found by the Wisniewski group to suppress Aβ‐related pathology in AD mouse models.^61^

Finally, an example of inhibitors derived from interaction partners are gramicidin S (GS) derived decapeptides; these were found by Abrahams and co‐workers to inhibit Aβ40 fibrillogenesis.^62^ Although GS is hemolytic, its analogues and other cyclic antibiotics could become promising anti‐amyloid drug candidates, as drug reprofiling is a fast way to reach the patient.^63^

### 4.4 Inhibitors Selected from Combinatorial Libraries

Various combinatorial peptide library approaches have been applied over the years (Figure 3).^20^ A good example is the all‐d linear dodecapeptide D3, which was identified early on by the Willbold group using mirror‐image phage and further developed over the past 10 years (Figure 6).44d D3 suppressed the amyloid self‐assembly of Aβ42 both in vitro and in AD mice models in vivo.44d Importantly, its optimized linear and cyclic analogues were recently found to exhibit BBB crossing ability and oral bioavailability in mice, which makes them promising AD anti‐amyloid drug candidates.^64^

## 5 Summary and Outlook

Here we discussed major molecular strategies and peptide chemistry tools to design, develop, and discover peptides as potent inhibitors of amyloid self‐assembly linked to the pathogenesis of numerous devastating neuro‐/cell‐degenerative diseases. We focused on two rational inhibitor design concepts: 1) based on molecular recognition principles of amyloid self‐assembly, the oldest and most common approach, and 2) based on cross‐amyloid interactions, a more recent approach. Their applicability was illustrated by discussing amyloid inhibitors of Aβ40(42), tau, IAPP, and insulin.

Together with small molecules and antibodies, peptides belong to the earliest developed anti‐amyloid molecules.^13^ However, very few of them, mostly earlier generations of linear peptides, reached (pre)clinical stages and, so far, none of them the patient.^13^, 14c

In principle, the failures of the clinical trials of all but one of the anti‐amyloid molecules could indicate that interfering with amyloid self‐assembly may not be the right disease‐modifying approach; in fact, this issue is currently under debate.^65^ However, compelling evidence still supports a key role of the amyloid formation process in disease pathogenesis.^4^ Thus, the multiple failures could also indicate that the drug candidate did not reach the target(s) in a timely and effective manner, as suggested for drug candidates for AD, which likely starts many years before the symptoms appear but remains undiagnosed.^65^ In addition, it is also possible that the tested agents—most of them highly promising antibodies and small molecules—simply did not meet the requirements of a disease‐modifying drug. Their consideration earlier in the process might improve the agent's prospects of reaching the clinic.

Such requirements could encompass:

1. a strong and broad inhibitor profile, that is, nanomolar inhibitory activity on all or many key microscopic steps of the cell‐damaging amyloid self‐assembly pathway; for example, as a consequence of the emerging crucial role of cell‐to‐cell transmission and (cross‐)seeding events, inhibitors with a broad, chaperone‐like function, including inhibition of secondary nucleation, could be required; alternatively, combinations of potent inhibitors with different conformation/species selectivities might be useful;^1^, 9a, 60b, 66
2. good drug‐like properties; and
3. in some cases good cell or BBB permeability.

Based on currently available molecular strategies and peptide chemistry tools and technologies, peptide‐derived inhibitors, in particular advanced generations, have the potential to fulfill many of the above requirements.^15^, ^16^ Such molecules would be promising anti‐amyloid leads or drug candidates for disease‐modifying treatments for AD and other cell‐degenerative diseases as well as valuable tools for deciphering the molecular, structural, and cellular basis of amyloid self‐assembly‐mediated cell damage and its link to disease.

## Conflict of interest

A.S. and A.K. are inventors on patent applications (A.S. and A.K.) or granted patents (A.K.) on some of the anti‐amyloid peptides included in this Minireview.

## Biographical Information

*Valentina Armiento obtained her B.Sc. in Chemistry in 2013 and her M.Sc. in Organic Chemistry in 2016 at Sapienza Università di Roma (Italy). In 2016 she started her PhD research in the group of A. Kapurniotu at the Technical University of Munich (TUM), where she investigates cross‐amyloid interactions and peptide‐based inhibitors of amyloid self‐assembly in AD and T2D*.

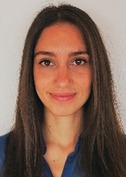

## Biographical Information

*Anna Spanopoulou studied Chemistry at the University of Ioannina in Greece and received her Diploma in 2010. She received her M.Sc. from the University of Patras in Greece at 2012. Her M.Sc. thesis concerned the synthesis of conotoxin analogues. She completed her PhD in the group of A. Kapurniotu at TUM in 2019. Her PhD studies focused on the design, synthesis, and study of conformationally constrained peptides as amyloid inhibitors*.

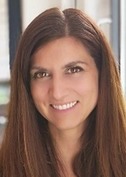

## Biographical Information

*Aphrodite Kapurniotu studied Chemistry in Athens and obtained her PhD in Tübingen in 1990. After postdoctoral research with J. W. Taylor at Rutgers University (1992–1994) as well as with R. Bucala and A. Cerami at the Picower Institute for Medical Research (USA; 1994–1995), she completed her Habilitation in Tübingen (2001) and moved to RWTH Aachen (2002). In 2007, she was appointed Professor for Peptide Biochemistry at TUM. Her research focuses on the development of peptide‐based leads for anti‐amyloid drugs and control of inflammatory chemokines in atherosclerosis*.